# Navigating the Collective: Nanoparticle-Assisted Identification of Leader Cancer Cells During Migration

**DOI:** 10.3390/life15010127

**Published:** 2025-01-19

**Authors:** Anastasia Alexandrova, Elizaveta Kontareva, Margarita Pustovalova, Sergey Leonov, Yulia Merkher

**Affiliations:** 1 The Laboratory of Personalized Chemo-Radiation Therapy, Institute of Future Biophysics, Moscow 141700, Russia; aleksandrova.av@phystech.edu (A.A.); leonov.sv@mipt.ru (S.L.); 2 Institute of Cell Biophysics of Russian Academy of Sciences, Pushchino 142290, Russia; 3 Faculty of Biomedical Engineering, Technion—Israel Institute of Technology, Haifa 3200003, Israel

**Keywords:** metastasis, cytoskeletal mechanisms, metastatic potential, endocytosis, nanoparticle encapsulation, actin filaments

## Abstract

Cancer-related deaths primarily occur due to metastasis, a process involving the migration and invasion of cancer cells. In most solid tumors, metastasis occurs through collective cell migration (CCM), guided by “cellular leaders”. These leader cells generate forces through actomyosin-mediated protrusion and contractility. The cytoskeletal mechanisms employed by metastatic cells during the migration process closely resemble the use of the actin cytoskeleton in endocytosis. In our previous work, we revealed that tumor cells exhibiting high metastatic potential (MP) are more adept at encapsulating 100–200 nm nanoparticles than those with lower MP. The objective of this study was to investigate whether nanoparticle encapsulation could effectively differentiate leader tumor cells during their CCM. To achieve our objectives, we employed a two-dimensional CCM model grounded in the wound-healing (“scratch”) assay, utilizing two breast cancer cell lines, MCF7 and MDA-MB-231, which display low and high migratory potential, respectively. We conducted calibration experiments to identify the “optimal time” at which cells exhibit peak speed during wound closure. Furthermore, we carried out experiments to assess nanoparticle uptake, calculating the colocalization coefficient, and employed phalloidin staining to analyze the anisotropy and orientation of actin filaments. The highest activity for low-MP cells was achieved at 2.6 h during the calibration experiments, whereas high-MP cells were maximally active at 3.9 h, resulting in 8% and 11% reductions in wound area, respectively. We observed a significant difference in encapsulation efficiency between leader and peripheral cells for both high-MP (*p* < 0.013) and low-MP (*p* < 0.02) cells. Moreover, leader cells demonstrated a considerably higher anisotropy coefficient (*p* < 0.029), indicating a more organized, directional structure of actin filaments compared to peripheral cells. Thus, nanoparticle encapsulation offers a groundbreaking approach to identifying the most aggressive and invasive leader cells during the CCM process in breast cancer. Detecting these cells is crucial for developing targeted therapies that can effectively curb metastasis and improve patient outcomes.

## 1. Introduction

In 2022, 2.3 million women were diagnosed with breast cancer (BC), resulting in 670,000 deaths worldwide [1]. Breast cancer occurs in every country; it can occur after puberty, with higher rates among older women, usually starting in milk ducts or lobules. Early-stage (in situ) cancer is not life-threatening, but metastatic cancer can be fatal. For instance, the 5-year survival rate for patients with triple-negative breast cancer (TNBC) is 77% [2], while it is only 7–10% for metastatic TNBC [3,4].

A key challenge in oncology diagnostics is accurately predicting the likelihood of metastasis. There are multiple robust methods available for this analysis: lymph node status, histology, tumor size, and gene expression analysis, along with methylation analysis [5,6]. It is important to note, however, that gene expression test systems are specifically applicable to a limited range of cancers that have well-defined genetic markers [7]. Moreover, the sensitivity of specific markers may fluctuate considerably based on the unique characteristics and comorbid conditions of a patient [8,9,10].

These nuances underscore the critical necessity of developing innovative methods capable of predicting metastasis in the early stages of tumor development, independently of genetics or biochemical markers. In a prior study, our laboratory established a novel quantitative approach to differentiating cancer cells exhibiting varying metastatic potentials, focusing on the efficiency of nanoparticle encapsulation by cells [11,12,13,14,15]. This method leverages the fundamental principle that the processes taking place in the cell cytoskeleton during migration and endocytosis share significant similarities.

Cell migration is a sophisticated process intricately linked to the actin-rich cortex located just beneath the plasma membrane. In numerous investigations focused on the relationship between endocytosis and the reorganization of cellular filaments during migration, several functional proteins have been highlighted: dynamin-1, eps-15, amphiphysin, Rho, Ras proteins, the WASp protein family, and mTORC2 [16,17,18,19,20,21,22].

During phagocytosis, the plasma membrane forms protrusions that reach out to capture particles, pulling them into the cell. This dynamic process is orchestrated by the coordinated actions of Cdc42 and Rac [23]. In a distinct form of phagocytosis, particles are drawn into invaginations lined with actin in the plasma membrane, with the process of internalization being reliant on RhoA [24]. The activation of Cdc42 on the inner membrane surface initiates actin polymerization and bundling, resulting in the formation of filopodia [25]. Similarly, the activation of Rac stimulates actin polymerization at the cell’s periphery, facilitating the development of sheet-like lamellipodial extensions [26]. The activation of Rho stimulates the assembly of actin filaments with myosin II filaments, resulting in the formation of stress fibers. Additionally, it encourages the clustering of integrins and related proteins, which leads to the development of focal adhesions [27].

Actin filaments are the primary drivers of cytoskeletal function across the cell body, displaying diverse architectures, such as the cortex, stress fibers, lamellipodia, and filopodia. These structures can be significantly influenced by the onset and progression of cancer, often leading to the adoption of new configurations as a consequence [28]. Significant differences in F-actin organization between non-malignant and invasive breast cancer cell lines have been revealed [29]. Additionally, investigation of the cell cortex, a delicate layer situated just beneath the plasma membrane, could provide valuable insights into the mechanisms of cancer development within cells. Comprising actin filaments, myosin motors, and actin-binding proteins, the cell cortex is believed to play a crucial role in determining cell stiffness [30,31]. Tabatabaei et al. [32] revealed intriguing disparities in actin anisotropy and migration between the benign MCF10A breast cell line and the malignant T47D line. Their findings indicate that the stress fibers in T47D cells extend in various directions, while the fibers in the normal MCF10A cells are aligned more uniformly along specific trajectories. One more compelling proof highlighting the significant role of and striking correlation between actin filaments in both migration and endocytosis is the impact of Latrunculin A on their disassembly. This effect has been demonstrated through the inhibition of endocytosis [33], as well as the impediment of migration and indentation in cancer cells [34]. Our research proposes that a common mechanism involving actin filaments operates during both cell migration and endocytosis. We believe that the efficiency of nanoparticle encapsulation may reveal significant insights into the migratory capacity of cells.

The migration of cancer cells represents a critical stage in the complex process of metastasis. This phenomenon can occur in various forms, including single-cell migration (which encompasses amoeboid and mesenchymal movements), multicellular streaming, collective cell migration, and expansive growth [35,36].

Clusters of metastatic cells have been demonstrated to be more invasive than individual cells [37], exhibiting significantly greater migration velocities [38] and facilitating the formation of metastases in vivo [39]. In most solid tumors, dissemination occurs via collective migration, orchestrated by leader cells, the “cellular leaders” [40,41,42,43].

A hallmark of cancer invasion is collective cell migration (CCM), which refers to the synchronized movement of groups of cells that maintain their connections with one another while effectively coordinating their actin dynamics and intracellular signaling processes [44].

The leader cells of the group leverage traction through the dynamic processes of actomyosin-driven protrusion and contractility, frequently working alongside sprouting cells located at the group’s periphery [45]. Consequently, the cells within the group may not directly engage with the extracellular matrix (ECM) at the leading edge; rather, they predominantly interact with adjacent cells and the intercellular matrix that forms along their junctions [40,41]. Akin to the collective cell migration observed during morphogenesis, the formation of cell–cell connections is likely enhanced by a variety of complementary adhesion systems [42]. These systems encompass cadherins, tight junction proteins, immunoglobulin superfamily adhesion receptors, and gap junctions. These components collaborate seamlessly to uphold mechanical cohesion among cells, create front-to-rear polarity within the group, synchronize the cytoskeleton, enable juxtracrine signaling, and potentially enhance mechanocoupling through desmosomes [43]. Recognizing cancer cells that could act as key drivers in metastatic spread is crucial.

The morphological organization of cancer cells during collective invasion can differ greatly. Clusters of invading cells may manifest as narrow strands of just one or two cells or as larger aggregates that encompass cells not directly connected to the extracellular matrix (ECM), and they can even develop luminal structures. This phenomenon is frequently observed in invasive carcinomas of the breast, prostate, and pancreas [44,46,47,48,49]. Cell adhesion and proteolytic activity affect the size and shape of invading structures. As a result, the leading edges of these cells can vary due to factors like proteolysis, protrusion, expansion, and the type of tissue involved [37,44]. In cancer cell invasion models, the leader cell usually has actin-rich protrusions that help it move forward by creating adhesive traction and breaking down the surrounding matrix [50]. Cell-followers play an important role in reinforcing this alignment, significantly increasing the diameter of the invading strand and enabling it to expand even further [43].

Leader cells play a crucial role in driving collective invasion in cancer [51], so identifying these cells provides potential therapeutic targets for cancer progression [52]. The leader cells can potentially be identified using various nanotechnology approaches, such as nanoparticle-based delivery systems and nano-diagnostic tools. For example, fluorescent and magnetic nanodiamond particles, which preserve the parental cell functions, have been applied for specific cancer cell labeling and tracking [53]. In addition, highly biocompatible chitosan- and ashwagandha-based nanoparticles were used for cancer cell labeling and early detection [54].

Cancer cells within the same tumor type can be classified into unique subpopulations by analyzing differences in their morphology, physical properties, gene expression, methylation patterns, and migratory abilities [55,56,57,58,59]. This study explores the variations among cell subpopulations based on their migratory activity. The efficiency of encapsulation of carboxylate-modified fluorescent nanoparticles by breast cancer cells with high metastatic (HM) potential and low metastatic (LM) potential has been investigated using the wound-healing assay, a well-established two-dimensional (2D) collective migration model. This assay enables the observation of the migration of confluent cells across a flat surface into an area made accessible by either wounding a cell monolayer or removing a barrier. We utilized commercial 200 nm FluoSpheres, which are carboxylate-modified microspheres infused with a green fluorescent dye. These microspheres proved effective in our previous efforts to differentiate between HM and LM tumor cells [14,60]. We have discovered a method of identifying leader cells serving as the leaders in collective migration that involves the use of quantitative high-content fluorescence detection techniques.

## 2. Materials and Methods

### 2.1. Cell Culture

The ATCC (American Tissue Culture Collection, Manassas, VA, USA) human MCF7 and MDA-MB-231 breast cancer cell lines were used in our study. ATCC reports that the doubling time for high-MP cells (ATCC CRM-HTB-26) is roughly 31 h, while low-MP cells (ATCC HTB-22) have a doubling time of about 29 h. The cell lines were cultured in their appropriate media, as recommended by ATCC: Dulbecco’s modified Eagle’s medium (DMEM) (Gibco, Thermo Fisher Scientific, Waltham, MA, USA) supplemented with 10 vol% fetal bovine serum (FBS) (Dia-M, Moscow, Russia), 1 vol% L-glutamine (Gibco, Grand Island, NY, USA), 1% antibiotics (100 U/mL penicillin, 100 µg/mL streptomycin) (Sigma-Aldrich, St. Louis, MO, USA), and sodium pyruvate (OOO NPP PanEco, Moscow, Russia). Cell lines were kept in a humidified atmosphere at 37 °C with 5% CO_2_. Cells were frozen at low passages from ATCC stock (i.e., 3–5), and for experiments, cells were thawed and used in passages 7–20 from the ATCC stock. Cell passage and culture conditions were identical for both cell lines. The various cell lines utilized in our experiments, along with the fetal bovine serum sourced from reputable suppliers, were thoroughly tested for the presence of mycoplasma using the same PCR assay, and the results consistently revealed a negative outcome, confirming the integrity of our experimental conditions.

### 2.2. Calibration Experiments

**Figure 1:**
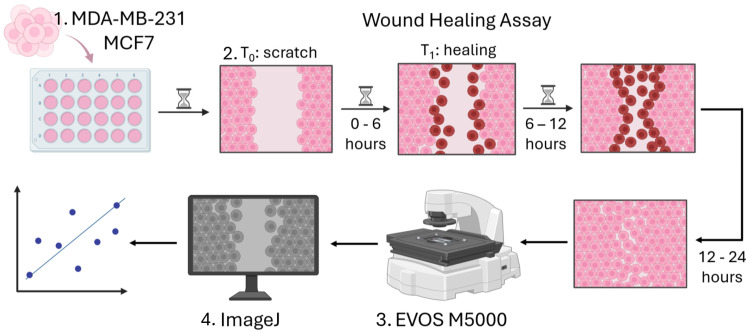
Graphical representation of calibration experiments of the in vitro wound-healing assay. This technique involves basic steps applicable to almost all cell types: (1) cell seeding and preparation; (2) making a linear thin scratch (creating a gap or “wound”) in a confluent cell monolayer; (3) acquiring data through microscopic imaging and measuring wound healing (gap closure) at each time point; and (4) data analysis.

The cells in normal growth medium were seeded into a 24-well cell culture plate. The cell-seeding concentration was 1 × 10^6^ cells/mL and allowed the formation of a monolayer. Once the cell monolayer was established, the medium was substituted with phosphate-buffered saline (PBS) (Gibco, Grand Island, NY, USA). A 20 µL pipette tip was then employed to make a scratch (wound) at the center of the well. Due to variations in wound width, the wound area at different time points was normalized to the initial wound area, A0. After washing off detached cells with PBS, we added DMEM supplemented with 5% FBS to mitigate proliferation, a commonly accepted procedure applied in wound-healing assays [61,62,63]. We first captured images of the monolayer wounds at the zero-hour mark and subsequently recorded hourly images along the wound edge for a total of 12 h. This allowed us to identify the “Optimal Time” (OT). Additionally, we took pictures after 24 h to assess the percentage of wound closure—effectively allowing the measurement of the migration efficacy endpoint—using the EVOS M5000 fluorescent imaging system (Thermo Fisher Scientific, Waltham, MA, USA) (Figure 1). To make z-stacks, we also used the real-time live cell imaging system JuLi Stage (NanoEntek, Seoul, Republic of Korea), which was installed in the incubator. The experiments were performed in triplicates, and 3 biological repeats were performed. To calculate the OT, we defined the maximal speed as “closure coefficient” and made calculations according to the following equation:

$$Closure\;coefficient=\frac{{Wound\;Closure}_{n-1}-{Wound\;Closure}_{n}}{∆t}$$

$${Wound\;Closure}_{n}=\left[\frac{A_{0}-A_{n}}{A_{0}}\right]\times 100\%$$

where A_0_ is the area of the wound measured immediately after scratching (0 h), and *An* is the area of the wound measured at *n* hours after scratching.

### 2.3. Nanoparticle Encapsulation and Actin Staining

**Figure 2:**
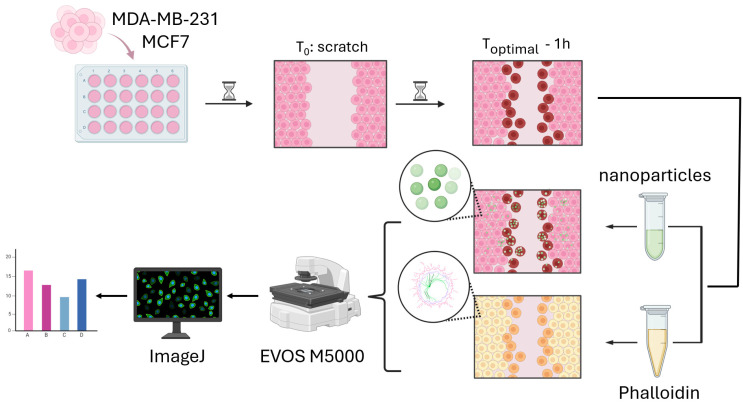
Graphical representation of workflow of nanoparticle encapsulation and actin staining during the in vitro wound-healing assay. This technique involves basic steps applicable to a wound-healing assay. For encapsulation experiments, 200 nm nanoparticles were added one hour before the OT. For experiments with actin markers, Phalloidin-iFluor 488 was added for 90 min following fixation of cells at OT.

We used yellow-green (excitation/emission: 505/515 nm) FluoSpheres Carboxylate-Modified Microspheres, particles 200 nm in diameter (Thermo Fisher Scientific, Waltham, MA, USA), to evaluate the adhesion and encapsulation efficiency of the cells (Figure 2). The viability of the cells incubated with the nanoparticles for 1 h and 24 h was above 92%, as determined via live/dead nuclei fluorescent staining [14]. We considered the results from the calibration experiments—the determined “optimal time” (OT) at which the cells attained their maximum movement speed. Nanoparticles were introduced one hour before reaching the OT for each cell line: for MCF7, this occurred at 1.6 h, while for MDA-MB-231, it was at 2.9 h. Considering that the doubling times of both cell lines significantly exceeded the observation period, no impact from the addition of nanoparticles on proliferation was detected. The final particle concentration of approximately 2000 particles/cell was chosen as optimal based on a comparison of the translocation coefficients of fluorescence intensity [14]. NucBlue™ Live ReadyProbes™ Reagent (Hoechst 33342) (Thermo Fisher Scientific, Waltham, MA, USA) was introduced to the cells 30 min prior to the conclusion of their incubation with nanoparticles, coinciding with the OT. The unbound nanoparticles were meticulously rinsed three times with PBS. Following this thorough washing process, no free-floating particles were observed [14]. Cells were fixed with 4% PFA (Paraformaldehyde) (PanEco, Moscow, Russia) and washed with PBS twice.

For staining filamentous actin (F-actin), Phalloidin-iFluor 488 Reagent was used (Abcam, Cambridge, UK) (Figure 2). At OT of wound healing, the cells were fixed with 4% PFA for 20 min. The staining was performed according to the manufacturer’s procedure. Briefly, Phalloidin-iFluor 488 was added for 90 min. NucBlue™ Live ReadyProbes™ Reagent (Hoechst 33342) (Molecular probes, Invitrogen life technologies, Carlsbad, CA, USA) was added for 30 min. Cells were washed three times with PBS. Cells were visualized using EVOS M5000 microscope (Invitrogen, Carlsbad, CA, USA) at 40× objective. At least 1500 cells per cell line were analyzed. The experiments were performed in triplicates, and 3 biological repeats were performed.

### 2.4. Microscopy and Imaging

Imaging was carried out with EVOS M5000 Imaging System (Thermo Fisher Scientific, Waltham, MA, USA), using a differential interference contrast (DIC) air-immersion, long-working-distance objective lens. The cells were maintained at 37 °C, 5% CO_2_, and high humidity (90%) throughout the entire experiment to sustain their viability. In the calibration experiments, the movement of cells was recorded every hour over a period of 12 h and again after 24 h using two different methods. The first method involved placing the cells in an incubator for 1 h and then removing them from the incubator for 20 min for photography using the EVOS M5000 Imaging System (Thermo Fisher Scientific, Waltham, MA, USA). The second method was conducted using the JuLI^TM^ Stage Real-Time Cell History Recorder (NanoEntek, Seoul, Republic of Korea) in Wound-Healing Assay mode. The JuLI^TM^ Stage was set up in the incubator, with the cells remaining inside for 24 h at 37 °C, 5% CO_2_, and high humidity (90%). A 10× phase contrast objective was used to assess the rate of wound closure. For the experiments involving nanoparticles and Phalloidin-iFluor 488, the EVOS M5000 Imaging System with a 40× objective was utilized using three channels: DAPI, GFP, and TRANS. The degree of light exposure was 0.002 for DAPI channel and 0.02 for GFP channel in each experiment. In each well, we imaged 9–10 randomly chosen fields of view. At each randomly chosen location, at least 3 images were taken.

ImageJ software (V1.53a, National Institutes of Health, Bethesda, MD, USA, and LOCI, University of Wisconsin, Madison, WI, USA) was utilized to determine the point at which cells attained their maximum speed during collective migration, assess the percentage of wound area, calculate the wound closure coefficient, measure area in µm^2^, evaluate standard deviation, and analyze the cell healing rate. We utilized the “Wound Healing Assay Tool” macros [64], configuring the parameters with precision: a variance window radius of 20, a threshold value of 20, and a percentage of saturated pixels set to 0.400, followed by selecting the appropriate stack. Leading cells were identified as those located right at the wound edge, specifically within 60 µm of it. We employed a custom-designed semi-automated co-localization macro [14], built upon ImageJ’s macros, to calculate the co-localization coefficient of fluorescent nanoparticles with respect to the imaged cells.

### 2.5. F-Actin Anisotropy and Orientation Analysis

**Figure 3:**
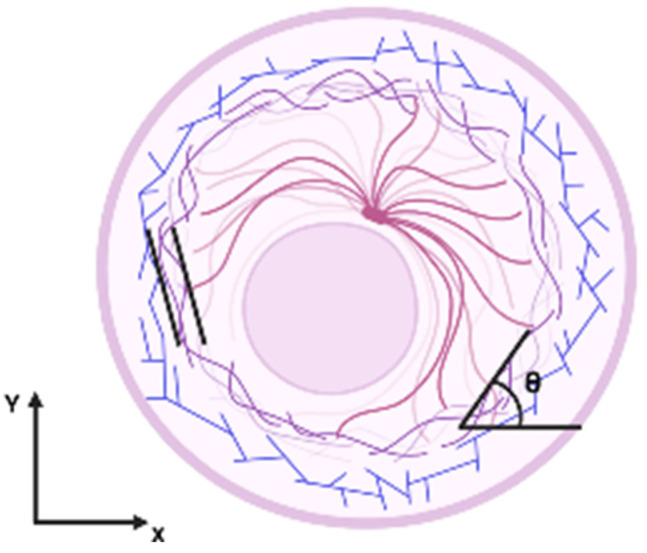
Schematic representation of the calculation of actin filament anisotropy and orientation. The diagram shows the structure of the cytoskeleton. Maroon indicates microtubules, while actin filaments are indicated in purple and blue. Black parallel lines show the way anisotropy is calculated. A coordinate system has been introduced. θ is the filament angle with respect to x. The orientation is determined by the angle—θ.

We conducted a comprehensive analysis of phalloidin experiments utilizing FibrilTool [65]. This macro allowed us to accurately calculate the anisotropy of the microfilaments and assess the angles of their deviation. The average angle and anisotropy of F-actin bundles were evaluated by analyzing the fluorescence signal from Phalloidin-iFluor 488 staining. Briefly, a 40× fluorescent image was opened in Fiji, and the region of interest was defined using the polygon tool, excluding areas with no signal. To calculate anisotropy and orientation, at least 16 cells were selected per image, with one area allocated for each cell, when the entire cell was selected. This macro calculates the average values for each cell. Next, we calculated the average value for each group of cells. The average orientation of fibers (−90° to 90°) and anisotropy (1 to 0) detected within the sample were recorded using the ‘FibrilTool’ function. This calculation is made based on using the concept of nematic tensor from the physics of liquid crystals to quantify the main orientation of fibrillar structures in an image and measure how well they are aligned. This tensor is computed from the pixel-intensity level in a region of an image. The gradient of intensity level enables the definition of a unit vector that is locally tangent to fibrils. The circular average of the tangent direction defines the average orientation in this region (fibril orientation), and the circular variance of the tangent direction defines the score assessing whether the fibrils are well ordered (fibril array anisotropy). This definition is equivalent to computing the nematic tensor. To define the anisotropy score, we used the following convention: 0 for no order (purely isotropic arrays) and 1 for perfectly ordered, i.e., parallel fibrils (purely anisotropic arrays). We calculated the orientation of actin filaments that shows the median angle of actin with respect to the horizontal axis. θ is the filament angle with respect to x. θ = 0° is the orthogonal direction to the wound. This means that even if the average angle shows a tendency towards positive or negative values, the cells exhibit a tendency towards a specific orientation. The orientation is determined by the angle-θ, as presented in Figure 3.

The anisotropy calculation was performed utilizing FibrilTool [65]. Briefly, the data were quantified as follows: if I (x, y) is pixel-intensity level in the image, as a function of the 2D coordinates (x, y), the unit vector

$$t=\left(t_{x},\;t_{y}\right)=\left(\frac{\partial I}{\partial y},-\frac{\partial I}{\partial x}\right)/\sqrt{(\partial I/\partial x{)}^{2}+(\partial I/\partial y{)}^{2}}$$

is the tangent of the putative fibrillar structures. The local nematic tensor n = t ⊗ t is the 2 × 2 symmetric matrix of components n _x_,_x_ = t_x_^2^, n _x_,_y_ = t_x_t_y_, and n _y_,_y_ = t_y_^2^. The nematic tensor of the region of interest (ROI) is the average < n > of the local tensor over the ROI. Let n_1_ > n_2_ be the eigenvalues of < n >. The eigenvector e_1_ of < n > corresponding to the eigenvalue n_1_ defines the main orientation of fibril array in the ROI, whereas q = n_1_ − n_2_ defines the anisotropy of the fibril array.

### 2.6. Statistical Analysis

Significance of variations between cell lines or cellular sub-populations were determined using the general linear mixed model, a univariate regression method for the analysis of variance (ANOVA), with a *p* value < 0.05. The Pearson correlation coefficient was used to determine colocalization of nanoparticles with the cells [14]. The calculations were performed using Excel (Microsoft, Redmond, WA, USA) and Python (Visual Studio Code) (Microsoft, Redmond, WA, USA).

## 3. Results

We used a wound-healing assay to study the collective migration of two BC cell lines: MCF7 and MDA-MB-231 (with low and high MP, respectively). Firstly, we conducted calibration experiments to find the “optimal time”, that is, when the cells reach the maximal speed during wound closure.

**Figure 4:**
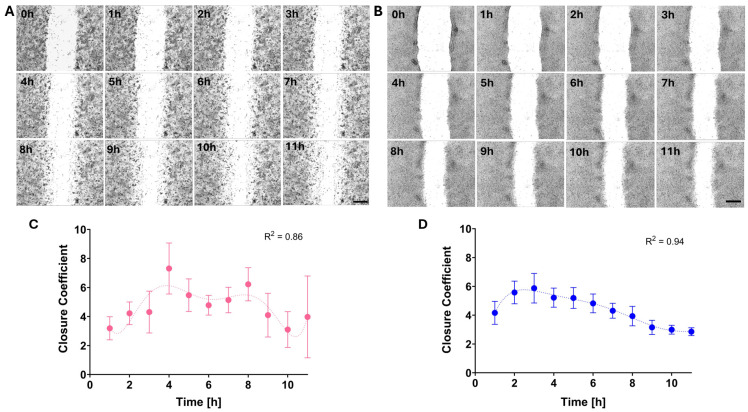
Images from a scratch assay experiment at different time points within 11 h. (**A**) MDA-MB-231. (**B**) MCF7. Cells were plated on plastic dishes, wounded with a pipette tip, and then imaged over 12 h using a microscope equipped with a point-visiting function and live-cell apparatus. Scale bar = 100 µm. (**C**,**D**) The dependence of the closure coefficient (in percentage) on time (in hours) for MDA-MB-231 and MCF7cell lines, respectively. Error bars are standard deviations.

We imaged the cells every hour for 11 h (Figure 4A,B). High-MP cells closed 76 ± 5% of the wound, while low-MP cells closed only 46 ± 6% of the wound. The highest wound closure coefficient was calculated as the local maximum for polynomial fit with adjustment coefficients (R^2^) of 0.86 and 0.94 for high- and low-MP cells, respectively (Figure 4C,D). The low-MP cells attained their peak speed after 2.6 h, while the high-MP cells reached theirs after 3.9 h, resulting in closures of 8% and 11% of the wound area, respectively. The significant deviation seen in low-MP cells at the 3-h time point and in high-MP cells at the 4-h time point may be explained by the activation of intracellular processes exclusively in a subset of highly active cells. The period during which the cells exhibited their maximal activity was designated as “optimal time” (OT) and subsequently used in experiments involving nanoparticle encapsulation. The maximal wound closure speeds observed at this OT were 9 ± 0.7 and 19.4 ± 3.1 µm/h for low- and high-MP cells, respectively. The average (for 11 h) wound closure speeds were significantly lower—5.3 ± 0.2 and 8.1 ± 0.7 µm/h for low- and high-MP cells, respectively. Extremely high heterogeneity of the high-MP cells was expressed by the high standard deviation at the 11 h time-point (Figure 4C), at which point total wound closure (closure coefficient = 0) was achieved for several experimental setups.

**Figure 5:**
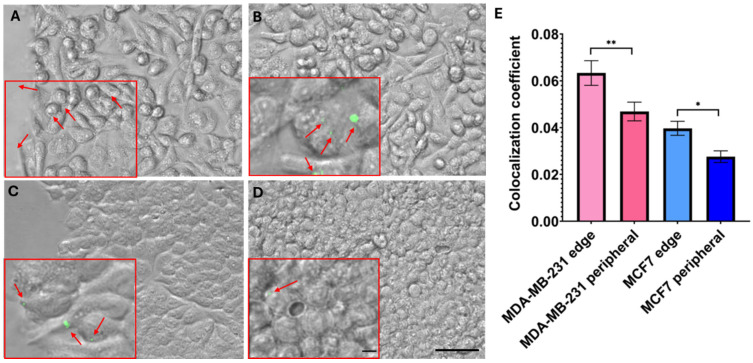
Internalization of carboxylate-modified 200 nm nanoparticles by BC cells. (**A**,**B**) High-MP cells on the leading edge and periphery, respectively. (**C**,**D**) Low-MP cells on the leading edge and periphery, respectively. Scale bar = 100 µm. The inserts in panels A-D display a chosen region of interest (ROI) magnified five times. The red arrows indicate the nanoparticles. Scale bar = 7 µm. (**E**) Colocalization of 200 nm nanoparticles with BC cells represented as the average value of Pearson coefficient in the edge and on the periphery of wound. The light- and dark-pink colors indicate leader and peripheral MDA-MB-231 cells, respectively; light blue—leader; dark blue—peripheral MCF7 cells. Statistically significant parameters: ** *p* < 0.013; * *p* < 0.02. Error bars are standard errors.

Subsequently, the cells were incubated with 200 nm fluorescent nanoparticles for one hour before the OT. The leader cells of both cell lines have more elongated morphologies and formed more protrusions (Figure 5A,C). The cells at the peripheral wound area (Figure 5B,D) have more-rounded morphologies and high confluency. The co-localization coefficients of nanoparticles associated with the cells (Figure 5E) were 0.063 ± 0.005 and 0.046 ± 0.004 at the edge of the wound and at the peripheral area, respectively, for cells with high MP. The colocalization coefficients for low-MP cells were significantly lower: 0.039 ± 0.003 and 0.027 ± 0.002 at the edge of the wound and at the peripheral area, respectively. We observed a significant difference in encapsulation efficiency between leader and peripheral cells for both high- and low-MP cells (*p* < 0.013) and significant differences between high and low-MP cells at the same wound area (edge/periphery), with *p* < 0.02.

**Figure 6:**
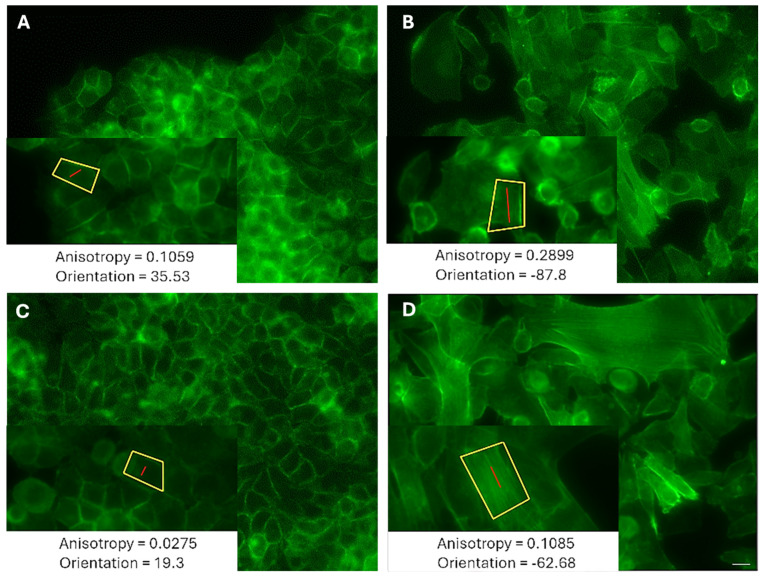
A typical image (selected FOVs × 40 magnification) of the phalloidin-stained actin fibers of LM (**A**,**C**) MCF7 and HM (**B**,**D**) MDA-MB-231 cells during migration. (**A**,**B**) Leader cells. (**C**,**D**) Peripheral cells. The inserts display a carefully chosen region of interest (ROI) magnified five times and taken while ensuring that no pixels became saturated. Output from FibrilTool: a line segment (red) is drawn, the angle of which represents the average orientation of the array and the length of which is proportional to the array’s anisotropy. The scale bar is 20 µm.

The morphologies of migrating cells with high and low MP are different. The leader low-MP cells (Figure 6A) exhibit a tightly packed and well-organized structure, resembling the cobblestone-like formation characteristic of epithelial cells. Their actin filaments show a low level of uniformity (anisotropy = 0.07), with a subtle inclination towards a specific direction (orientation = −1.04), evident in the overall arrangement of the cells. The peripheral low-MP cells (Figure 6C) also exhibit a seemingly random orientation (−1.06) within a tightly packed configuration, devoid of any discernible directional structure. Their minimal alignment (anisotropy = 0.06) indicates that both the cells and their actin stress fibers are positioned in multiple directions with no dominant preference, resulting in a chaotic, isotropic appearance. In contrast, the leader high-MP cells (Figure 6B) display a more dispersed arrangement, characterized by significant gaps or voids between the cells, indicating a less dense structure. These cells display clear alignment (anisotropy = 0.18), evident in their elongated shapes that follow a specific orientation (2.5). Similarly, the peripheral high-MP cells (Figure 6D) exhibit elongated and aligned forms (anisotropy = 0.16), taking on a spindle-like shape that suggests pronounced alignment along a distinct axis (orientation = −8.6).

**Figure 7:**
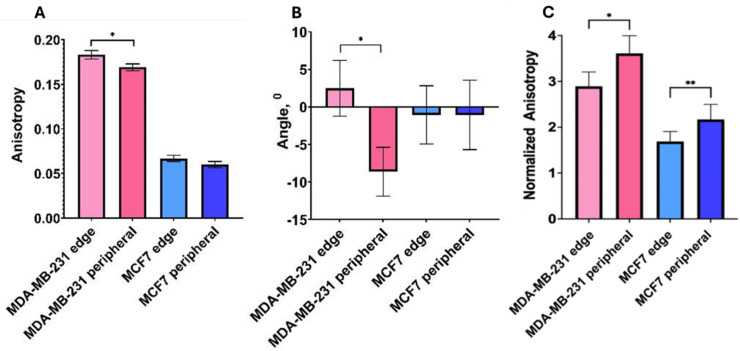
Anisotropy (**A**) and orientation (**B**) obtained for all tested cell types.; *—*p* < 0.02. (**C**) Anisotropy normalized by the Pearson colocalization coefficient. *—*p* < 3 × 10^−10^; **—*p* < 0.003. The light- and dark-pink colors indicate data for leader and peripheral MDA-MB-231 cells, respectively; light blue and dark blue indicate data for leader and peripheral MCF7 cells, respectively. Error bars are standard errors.

A quantitative analysis of the properties of F-actin filaments in migrating breast cancer cells with varying metastatic potentials revealed no significant differences between leader and peripheral cells in the low-MP group, with comparable results regarding anisotropy (*p* = 0.165) and directionality (*p* = 0.996). In the high-MP cell line, significant statistical differences were observed in anisotropy (*p* < 0.029) and filament directionality (*p* < 0.024), as demonstrated in Figure 7A,B. Notably, the anisotropy values for the high-MP cells were 2.7 to 2.8 times greater than those for the low-MP cells at both the leading edge (leaders) and the peripheral area. To correlate the cytoskeletal organization with uptake properties in a quantitative manner, we normalized the filaments’ anisotropy to encapsulation ability. The results indicate that the normalized coefficients are significantly higher for peripheral cells for both cell lines (*p* < 0.003), whereas for low-MP cells, the normalized coefficients are significantly lower (*p* < 0.0001) (Figure 7C).

## 4. Discussion

Studies on metastatic activity have focused on two key breast cancer cell lines: the highly invasive MDA-MB-231 line and the low-level-invasive MCF7 line. Research shows both similarities and differences between these cell lines and highlights the heterogeneity in MDA-MB-231 cells. Many studies link the aggressiveness of cancer cells to their wound closure speed.

Our research reveals that after 11 h, high-motility-potential (MP) cells are capable of closing a 1.65-times-greater area compared to low-MP cells under identical conditions. After 24 h, however, wound closure rates were comparable between high- and low-MP cells, achieving 93 ± 3% and 91 ± 5% closure, respectively. This finding underscores the differences in cell migration speed during the closure process. Consistent with our findings, the MDA-MB-231 cells demonstrated a significant 40% wound closure within just 5 h, achieving total closure by the 72-h mark. In contrast, the less invasive MCF7 cells only managed 40% closure after 10 h [66]. A recent study [67] demonstrated that both cell lines attained comparable wound closure rates over a 24-h period, reaching 65–70%. In contrast, in a microfluidic device, both cell lines completely closed wounds within 24 h, achieving an average migration speed of 13 μm/h. Additionally, in this study, a comparable number of cells migrated through the Boyden chambers following the onset of cell starvation conditions [68], a finding that is contradictory to our previous study [34]. Yet another study [69] revealed striking discrepancies in wound closure rates, with MCF7 healing at a rate of 7 μm/h, which can be compared to MDA-MB-231’s 33 μm/h, over a 24-h period. In a study examining cell invasion into Matrigel [70], MDA-MB-231 cells exhibited a notable average invasiveness rate of 2.7 μm/h over a five-day period. In contrast, their migration rate in microfluidic chips jumped to 6.7 μm/h, significantly outpacing the migration rate of the benign MCF10A cell line, which was recorded at 2.1 μm/h. Thus, investigating the migratory activities of these cell lines in 2D systems yields a variety of data, influenced by experimental conditions and the inherent heterogeneity of the cell lines. Consequently, these studies do not consistently yield clear conclusions about the connection between migratory capabilities and the aggressiveness of cancer cells. Hence, it is crucial to identify the cells that drive the migration process, as this plays a vital role in tissue development, regeneration, and various pathologies, including tumorigenesis. Leader cells, which are pivotal cells in collective migration, are a significant focus of research.

In a two-dimensional wound model, the cells at the wound’s edges undergo transformation from an epithelial to a mesenchymal phenotype. As a result, their shape shifts from a rigid structure to a fluid, amoeboid-like form [71,72]. During this phase, the cells adjacent to the posterior region increasingly become more fluid while preserving their intercellular connections and enhancing the formation of focal adhesions. The morphology of cells plays a crucial role in determining their metastatic potential [73]. Consequently, the distinctive shape of leader cells during collective migration allows them to be identified separately from the overall cell population [74]. Leader cells exhibit distinctive morphological features, including an elongated shape and lengthy outgrowth. Biochemically, they show heightened expression of proteins linked to motility and focal adhesion while simultaneously reducing the expression of intercellular adhesion proteins [75].

Anisotropy is a numerical measure that reflects the extent of directional dependence within a structure. Elevated anisotropy values denote a higher degree of organization and directional alignment among cells or cellular components, whereas lower values signify a more random orientation. Our findings revealed that high-MP cells exhibited more than three times the organization in anisotropy compared to low-MP cells. Numerous studies have documented cancer-induced alterations in the organization of actin stress fibers; however, the reported extent and patterns of these changes vary significantly across the literature. A number of studies have demonstrated a correlation between cancer and a decrease in actin content. For instance, the disruption of actin filaments by latrunculin A nearly halted the indentation of breast cancer cells with high metastatic potential [34]. Conversely, certain cell lines have demonstrated a contrasting trend [76]. A pioneer study comparing tumorigenic (HeLa parental cell line) and non-tumorigenic (HeLa–fibroblast fusion hybrid) cells revealed distinct differences in microfilament organization and a substantial decrease in actin concentration in the tumorigenic cells, whether assessed on a per cell basis or per protein. Notably, the tumorigenic cell lines demonstrated a 35% reduction in actin content compared to their non-tumorigenic counterparts, regardless of cell density [77]. A study comparing normal breast cells with invasive cancer cell lines demonstrated a notable decrease in the cell actin index, indicating a reduction in the cell cortex within suspended cancerous cells [32]. Additional research indicates that cancer initiation and progression result in a decreased level of cytoskeletal actin [78,79].

Typically, a reduction in actin content would be expected to lead to decreases in cellular activities and properties such as motility; however, in metastatic cancer cells, this reduction paradoxically correlates with enhanced motility and substantial contractile forces. This paradoxical behavior can be understood from two perspectives: the first relates to the alterations in the fibrous arrangement and the orientation of actin structures that improve cell motility, even with a reduced actin content, and the second concerns the significance of actin-binding proteins in this dynamic process. Actin structure remodeling takes place during both the initiation and invasion phases of cancer [80]. Research indicates that the remodeling of actin structures plays a crucial role in the process of epithelial-to-mesenchymal transition (EMT) [81]. While tumorigenic cells exhibited an increased number of stress fibers, it was concluded that a disorganization of actin structures serves as a key indicator of the progression from low-level-invasive to highly invasive and metastatic malignant cells [82]. Our findings reveal that the anisotropy coefficient is elevated in leader high-MP cells. Consequently, in cells characterized by high MP, there is an increase in the formation of actin stress fibers, which enables us to infer their enhanced ability to metastasize. Orientation plays a crucial role in dictating the directionality of stress fibers and their relative arrangement, specifically in the positioning of parallel-oriented actin filament sets within the cell. Comparable outcomes regarding the unidirectional alignment of actin fibers were noted following the treatment of HeLa cells [83]. A greater diversity in angle values correlates with an increased presence of multidirectional stress fibers within the cell. This indicates that these cells exhibit enhanced migratory activity, which in turn elevates their metastatic potential.

A previously established and validated method offers a straightforward and effective means of distinguishing cancer cells with differing metastatic potentials [11,14,15]. Utilizing the same approach, we successfully distinguished between leader and peripheral BC cells. Our research revealed that leader cells from both cell lines are capable of encapsulating 200 nm particles 1.4 times more efficiently than peripheral cells of the same type. However, the normalized-by-encapsulation-capacity anisotropy coefficient in peripheral cells is higher than in leader cells. This coefficient is statistically different for both cell lines, revealing both anisotropy and encapsulation ability. This finding underscores the significant heterogeneity of breast cancer (BC) cells within a single cell line. For instance, BC cells derived from the same cell line exhibit varying levels of E-cadherin, progesterone, and estrogen receptors, as well as fluctuations in Her2 receptor expression [84,85]. Research has demonstrated that only a specific percentage of cells from the same cell line population are capable of invading [86] or migrating [87] in vitro. This heterogeneity enables cancer cells to adapt their shapes and behavior in response to the microenvironment, allowing them to utilize more aggressive cells for invasion and migration, ultimately facilitating the development of metastases.

The unique characteristics of these leader tumor cells not only facilitate their migratory capabilities but also distinguish them from surrounding cells. This differentiation highlights their significance as prime targets for developing effective anti-metastatic therapies. It was found that ovarian cancer leader cells were resistant to a variety of chemotherapy drugs and could play a crucial role in the recurrence of chemotherapy-resistant disease, contributing to unfavorable treatment outcomes [88]. Additionally, it was shown that breast cancer leader cells can be randomly distributed in a tumor and determined by K14+ expression following a reaction to chemical and mechanical stimuli via polarization [89]. Nanotechnology has groundbreaking potential in revolutionizing cancer treatment, especially with the integration of advanced nanoparticle-based delivery systems and innovative nano-diagnostic tools. Nanoparticle-based delivery systems can precisely target cancer cells, enhancing the effectiveness of chemotherapy, targeted therapy, and immunotherapy while minimizing damage to healthy tissues [90]. These systems can overcome drug resistance by targeting specific mechanisms within cancer cells [90]. Nano-diagnostic tools offer advanced methods for early cancer detection and monitoring. These tools can detect cancer biomarkers with high sensitivity and specificity, enabling earlier and more accurate diagnoses [91]. Additionally, they can track the progression of cancer and the effectiveness of treatments in real time [91]. Targeting leader cells—a subpopulation of cancer cells that drives collective invasion and metastasis—using nanotechnology is an emerging area of research. Nanoparticles can be designed to specifically target these cells, potentially inhibiting their role in metastasis and improving treatment outcomes [92].

## 5. Conclusions

In conclusion, our research highlights the crucial importance of nanoparticle encapsulation in pinpointing leader cells during the collective migration of breast cancer cells. These leader cells, recognized for their heightened metastatic potential, play a crucial role in the metastasis process, which is the primary cause of cancer-related fatalities. Utilizing nanoparticles allows us to effectively differentiate aggressive cells from their less-invasive counterparts. Our research demonstrates that leader cells possess superior encapsulation efficiency and increased anisotropy in their actin filaments when compared to peripheral cells. This indicates that the cytoskeletal dynamics of leader cells are unique and can be utilized for their identification. While our previous work [14] laid the groundwork, it did not differentiate between cell sub-populations within the same cell line. This study advances this field by providing a more detailed analysis of leader cell behavior and its correlation with metastatic potential.

Accurately recognizing these cells is crucial for creating targeted therapies aimed at specifically hindering the metastatic potential of cancer cells. This advancement could significantly diminish cancer spread and enhance patient outcomes. Future research should improve nanoparticle techniques and thus facilitate better accuracy and reliability in clinical use. It should also investigate how these findings can be applied in treating cancer more effectively. Focusing on the most aggressive cancer cells allows us to create strategies that are not only more targeted but also significantly more effective in fighting metastasis. This approach ultimately leads to improved prognosis and survival rates for cancer patients.

## Data Availability

The raw data supporting the conclusions of this article will be made available by the authors on request.
